# Midwives’ perspectives on (dis)respectful intrapartum care during facility-based delivery in sub-Saharan Africa: a qualitative systematic review and meta-synthesis

**DOI:** 10.1186/s12978-019-0773-y

**Published:** 2019-07-25

**Authors:** Susan Bradley, Christine McCourt, Juliet Rayment, Divya Parmar

**Affiliations:** School of Health Sciences, City, University of London, 1 Myddelton Street, London, EC1R 1UW UK

**Keywords:** Midwifery, Disrespect and abuse, Childbirth, Sub-Saharan Africa, Respectful maternity care, Facility-based delivery

## Abstract

**Background:**

In the past decade, the negative impact of disrespectful maternity care on women’s utilisation and experiences of facility-based delivery has been well documented. Less is known about midwives’ perspectives on these labour ward dynamics. Yet efforts to provide care that satisfies women’s psycho-socio-cultural needs rest on midwives’ capacity and willingness to provide it. We performed a systematic review of the emerging literature documenting midwives’ perspectives to explore the broader drivers of (dis)respectful care during facility-based delivery in the sub-Saharan African context.

**Methods:**

Seven databases (CINAHL, PsychINFO, PsychArticles, Embase, Global Health, Maternity and Infant Care and PubMed) were systematically searched from 1990 to May 2018. Primary qualitative studies with a substantial focus on the interpersonal aspects of care were eligible if they captured midwives’ voices and perspectives. Study quality was independently assessed by two reviewers and PRISMA guidelines were followed. The results and findings from each study were synthesised using an existing conceptual framework of the drivers of disrespectful care.

**Results:**

Eleven papers from six countries were included and six main themes were identified. ‘Power and control’ and ‘Maintaining midwives’ status’ reflected midwives’ focus on the micro-level interactions of the mother-midwife dyad. Meso-level drivers of disrespectful care were: the constraints of the ‘Work environment and resources’; concerns about ‘Midwives’ position in the health systems hierarchy’; and the impact of ‘Midwives’ conceptualisations of respectful maternity care’. An emerging theme outlined the ‘Impact on midwives’ of (dis)respectful care.

**Conclusion:**

We used a theoretically informed conceptual framework to move beyond the micro-level and interrogate the social, cultural and historical factors that underpin (dis)respectful care. Controlling women was a key theme, echoing women’s experiences, but midwives paid less attention to the social inequalities that distress women. The synthesis highlighted midwives’ low status in the health system hierarchy, while organisational cultures of blame and a lack of consideration for them as professionals effectively constitute disrespect and abuse of these health workers. Broader, interdisciplinary perspectives on the wider drivers of midwives’ disrespectful attitudes and behaviours are crucial if efforts to improve the maternity care environment - for women and midwives - are to succeed.

## Plain English summary

Disrespect and abuse of women during labour and delivery is a major public health concern. There is considerable evidence documenting women’s unhappiness with care that does not satisfy their need for social, emotional and psychological safety. We also know that women value and benefit from respectful maternity care. However, less is known about midwives’ perceptions of labour ward dynamics or why they think disrespectful care happens. To address this, our team searched for published studies that explicitly captured midwives’ voices. We only included papers if they directly reported midwives’ experiences of the interpersonal aspects of facility-based delivery.

Our review showed that it is important to look beyond the immediate relationship between the woman and the midwife in the labour ward, as a range of upstream factors influence the way that interpersonal care is delivered. The most common issue midwives reported was the challenge of having too few midwives or resources to be able to spend time with women and to meet professional standards. At the same time, midwives were aware that they were considered to have low status in the health system hierarchy, leaving them feeling unvalued and blamed when things went wrong. These were powerful drivers of some of the disrespectful behaviours midwives described. We concluded that it is crucial to have a broader gaze on the factors driving disrespect if effective changes are to be made to improve the birthing environment for both women and midwives.

## Background

Policies to increase the rate of facility-based delivery have been a central pillar of the international community’s efforts to improve maternal and neonatal health [1]. Despite significant efforts, maternal mortality ratios in sub-Saharan African countries remain high; in 2015, the region bore 66.3% of the global burden of maternal deaths [2]. Rapid increases in the number of women birthing in health facilities have occurred ahead of improvements in the capacity of health systems to accommodate these, resulting in low quality of care [3]. Sub-Saharan Africa’s colonial past is relevant here. It has significant implications for the history and shape of health systems [4], while the impact of debt and strict austerity measures continues to compromise the functioning of these systems [5]. The effects are felt most keenly at the primary health care level where the majority of facility-based deliveries occur.

Midwives are key frontline staff at the primary level and the challenges facing them are considerable. Shortages in absolute numbers or maldistribution of the existing midwives (e.g. [6, 7]) are a serious constraint. These combine with harsh work environments, characterised by inconsistent supplies of basic commodities and supplies, to make it difficult for staff to provide optimum care [8]. These factors result in a perfect storm, where the challenges of the health system, increasing numbers of facility-based deliveries, poverty and lack of resources, collide in the labour ward, making power issues more visible, but also interact with existing inequalities to exacerbate the power dynamics at play.

Substantial evidence of women’s unhappiness with their experience of care has raised awareness of disrespect and abuse (D&A) of women during birth as a significant global public health issue. Bohren et al.’s [9] review of women’s experiences of facility-based delivery demonstrated women’s perceptions that birth had become medicalised and dehumanised. Further reviews have: provided insights into aspects of health worker behaviour affecting women’s satisfaction and wellbeing [10, 11]; expanded the typologies of D&A as mistreatment and included the role of systemic health systems failures [12]; and highlighted the consistency across countries on women’s views of what constituted respectful maternity care (RMC) [13]. Throughout, a clear picture has emerged of a routine lack of attention to the sociocultural and psycho-emotional salience of birth and the ways in which this intersects with structural inequality to manifest in behaviours that do not satisfy women’s needs [14].

Recent global shifts in attitudes to maternity care provision have recognised the international community’s ‘blind spot’ to the quality dimension of respectful, woman-centred care, along with the over-medicalisation of childbirth [15]. This was highlighted in the Lancet’s Series on Midwifery [16], which provided a high-profile, critical examination of global midwifery. The Series articulated key concerns, such as the importance of midwives’ attitudes and interpersonal/cultural competence, and the imperative to normalise biological, psychological, social and cultural processes. These were all set within the context of respectful care, where midwives should work in partnership with women and strengthen women’s capabilities. These recommendations were based on what women need and want [17], marking the recognition that quality midwifery care is not only about the provision of care, but, crucially, also about how it is experienced [18, 19]. This represents a shift away from a false, and sometimes oppositional, separation of safety from normality and humanised care [20, 21]. International ambitions for a more woman-centred model of care [22–25] were recently encapsulated in the WHO’s Recommendations on Intrapartum Care for a Positive Childbirth Experience [26].

A key element of providing more holistic care that addresses women’s psycho-socio-cultural needs is the capacity and willingness of midwives to provide it. Yet, until very recently, there was limited exploration of midwives’ perspectives and their voices were largely excluded from the discourse. An additional limitation has been the tendency in much of the D&A literature to focus in on the micro-level interaction of the midwife-woman dyad. This reflected an implicit, but now changing, assumption that things would improve if only midwives would be kinder, nicer, more professional. Such an approach neglected the reality in which midwives’ behaviour was embedded and the impact of broader historical, cultural and social factors. The focus has recently shifted and there is now growing recognition that a broader gaze is needed to understand the factors that affect labour ward dynamics. Work by Filby et al. [27] expanded the debate, highlighting the intersecting social, economic and professional barriers midwives faced in providing quality care, barriers which stemmed from gender inequality and caused significant burnout and moral distress. In 2016, our team examined women’s perspectives on disrespectful intrapartum care during facility-based delivery in sub-Saharan Africa [14]. We explored macro-, meso- and micro-level drivers, remaining aware of the interplay of the postcolonial context, structural inequality, and health system policy and drivers. The review was completed in late-2015, at a time when there was very little literature exploring the perceptions of midwives on the interpersonal elements of care. In that review, we used women’s experiences as the lens through which to explore the drivers of disrespectful care, to try and understand what caused midwives to behave in the manner that women reported. By early-2017, however, a small body of mainly descriptive studies had begun to emerge, documenting midwives’ perspectives on the interpersonal elements of facility-based delivery in sub-Saharan Africa. A second systematic qualitative review, presented here, was undertaken to synthesise this literature, using midwives’ voices and perspectives to explore the broader drivers of (dis)respectful care during facility-based delivery.

## Methods

### Searching and screening

The Preferred Reporting Items for Systematic Reviews and Meta-Analyses (PRISMA) guidelines [28] were used as a checklist for the search and screening stage of the review. Searches were carried out by SB in: CINAHL, PsychINFO, PsychArticles (all EBSCO platform); Embase, Global Health, Maternity and Infant Care (all OVID platform); and PubMed, to identify eligible papers published between 01/01/1990–16/02/2017. ‘Cited by’, ‘related citations’ and manual searches of reference lists for each included publication were carried out and searches were updated in May 2018. An example of the search strategy is provided in Table 1.

**Table 1:** PubMed search strategy

Midwives’ experiences	1.	nurse* OR provider* OR health worker* OR sage*femme* OR “skilled birth” OR “midwifery”[MeSH] OR “nurse midwives”[MeSH]
	2.	experience* OR perception* OR view* OR opinion* OR attitud* OR perspective* OR belie* OR account* OR narrative* OR story OR stories OR distress OR emotion* OR moral* OR ethic*
	3.	1 AND 2
Birth	4.	“Delivery, Obstetric”[Mesh]) OR “Perinatal Care”[Mesh]) OR “Parturition”[Mesh]) OR “Labor, Obstetric”[Mesh] OR childbirth* OR birth* OR deliver* OR labour OR labor OR “maternity care” OR “intrapartum care” OR “obstetric care”
Interpersonal care	5.	“quality of care” OR respectful matern* OR support* OR respect* OR disrespect* OR abus* OR caring OR violen* OR digni* OR neglect* OR psychosocial OR relationship* OR mistreatment OR interpersonal
Sub-Saharan Africa	6.	“Africa South of the Sahara”[Mesh] OR Burundi OR Djibouti OR Eritrea OR Ethiopia OR Kenya OR Rwanda OR Somalia OR Sudan OR Uganda OR Tanzania OR Benin OR “Burkina Faso” OR “Cote d’Ivoire” OR Gambia OR Ghana OR Guinea OR Guinea-Bissau OR Liberia OR Mali OR Mauritania OR Niger OR Nigeria OR Senegal OR “Sierra Leone” OR Togo OR Cameroon OR “Central African Republic” OR Chad OR Congo OR “Democratic Republic of the Congo” OR “Equatorial Guinea” OR Gabon OR Angola OR Botswana OR Lesotho OR Malawi OR Mozambique OR Namibia OR “South Africa” OR Swaziland OR Zambia OR Zimbabwe OR “Cape Verde” OR Comoros OR Madagascar OR Mauritius OR Mayotte OR Reunion OR “Sao Tome and Principe” OR Seychelles
Full search	7.	3 AND 4 AND 5 AND 6

Filter: From 1990

All terms were searched as Title/Abstract, except MeSH headings

Table 2 shows the inclusion/exclusion criteria. Included studies were based in sub-Saharan Africa and had a substantial qualitative element exploring the perspectives of midwives or nurse-midwives, working in maternity wards, on the interpersonal aspects of intrapartum care. Our inclusion criteria were practicing midwives currently engaged in facility-based delivery. In common with other authors (e.g. [29]) however, we found a lack of clarity on qualifications or levels of training. Papers were considered if it was clear that they included qualified midwives who were based in labour wards or health facilities and were responsible for conducting deliveries. Those outside of these settings, or where their qualification was not licensed or accredited, such as some auxiliary midwives, were excluded. Midwifery students were also included as their training involves significant clinical practice in the labour ward. A key aim was to foreground the missing voice of the midwife, so only publications directly reporting midwives’ views were eligible for inclusion. All retrieved items were screened by SB using title/abstract to exclude clearly irrelevant items. Full texts of all potentially relevant items were screened by SB and two other members of the review team (JR and DP). Only references that satisfied all three reviewers were included.

**Table 2:** Inclusion and exclusion criteria

	Inclusion	Exclusion
Participants	Midwives, nurse-midwives or midwifery students^a^ working in maternity wards/units and carrying out facility-based delivery	Midwives working outside health facilities or where the site of delivery is unclear Specific focus on perinatal loss, severe maternal morbidity or HIV
Intervention	Midwives’ views, perceptions and experiences of the interpersonal aspects of facility-based intrapartum care, or the impact of this element of care	Ante- or post-natal care only Clinical or technical quality of care only Midwives’ experiences described by other actors (e.g. women, community members)
Outcomes	Any	N/A
Study design	Primary qualitative studies (IDI, FGD) including, but not limited to, designs such as phenomenology, grounded theory, ethnography, action research and feminist research, or mixed-methods studies with a relevant qualitative element	Quantitative studies, RCTs, quantitative findings from mixed-methods studies Open-ended questions in survey-based studies
Study focus	Midwives’ experience and perceptions of (dis)respectful care either as the main focus of the study or as a substantial element of it	Main focus is not on midwives’ perceptions of intrapartum care Focus is specifically on technical aspects of care
Setting	Sub-Saharan Africa, including Sudan	Algeria, Egypt, Libya, Morocco, Tunisia and Western Sahara
Time period	1/1/1990–05/05/2018	Pre-1990
Language	Only abstracts available in English, French or Portuguese will be assessed	None
Publication type	Peer reviewed articles, dissertations/theses or research reports	Reviews, opinion pieces, policy documents

^a^Collectively referred to as ‘midwives’

### Quality appraisal

Two reviewers independently assessed the methodological rigour of all included studies using the Critical Appraisal Skills Programme tool for qualitative research [30]. Studies were rated high, medium or low quality for each domain and assigned an overall quality score. However, study quality was not used to exclude studies with the potential to answer the review question.

### Data extraction and synthesis

**Fig. 1:**
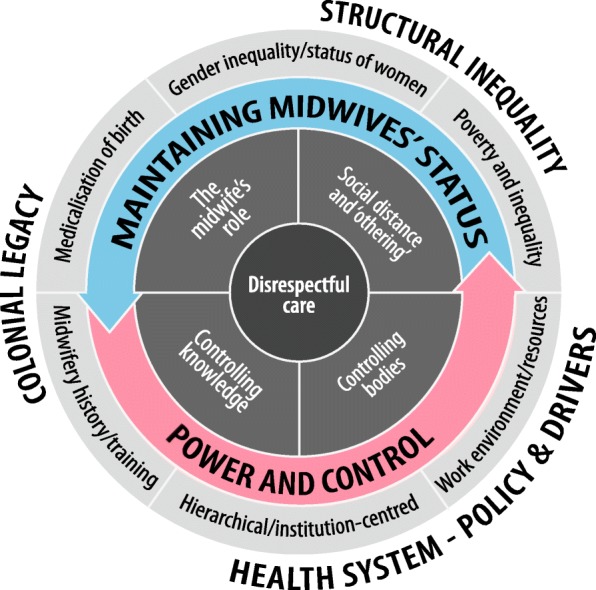
Conceptual framework of the drivers of (dis)respectful care in the sub-Saharan Africa context [14]

The results section of each study paper, including participant quotes, was imported in full and verbatim by SB into NVivo 11 software for data analysis. Our previous review of women’s experiences [14] used Thomas and Harden’s [31] thematic synthesis method, which allowed us to develop analytical themes and bring fresh interpretations. These synthesis results were used to develop an original conceptual framework of the drivers of (dis)respectful care in the sub-Saharan African context (Fig. 1.) which we have used in the review presented here to analyse midwives’ experiences. The conceptual framework describes how micro-level interactions in the labour ward are mediated by meso- and macro-level influences. In the model, the flow of influence is from the outside to the centre, situating disrespectful care within a broader framework of the structural dimensions underpinning disrespect that are often neglected in discussions of the mistreatment of women.

Two overarching analytical themes are ‘Power and control’ and ‘Maintaining midwives’ status’. ‘Power and control’ describes midwives’ attempts to exert control over women and the birth process. Controlling women’s bodies and how they physically behave during labour focuses on the trigger points of expressions of pain and the timing and direction of the pushing stage of labour. Controlling women’s knowledge includes two elements. Firstly, authoritative knowledge^1^ [32], where a woman’s embodied knowledge of what her body needs to do is overridden; and secondly, withholding information so that women do not know what is happening. Together, these controls relegate women to the role of bystander, not participant, in the birth. Control is achieved using various forms of discipline and punishment. The second major theme is ‘Maintaining midwives’ status’, where midwives attempt to maintain their own professional, technical and social status by reinforcing the social distance between themselves and the women in their care. The strategies they use to do this are grouped into two main themes. One covers decisions about what constitutes the midwife’s role, with an emphasis on the technical care during the second stage. The other describes midwives’ attempts to maintain status through social distancing and ‘othering’, using social inequality, sexual shaming, and an unwillingness to do ‘dirty work’^2^ [33, 34].

For the synthesis presented here, a coding framework was constructed using the individual domains of our conceptual framework as top-level nodes at the macro-, meso- and micro-levels. Line-by-line coding of the findings of each paper allowed data relevant to the domains to be captured, while any data that did not fit the framework were inductively free-coded into new nodes. Three papers were independently coded by reviewers [SB, CM, DP] to identify themes arising and to assess how well these mapped onto the framework. This facilitated a transparent and flexible process where convergence or divergence between the insights gleaned from women’s experiences and those of midwives could also be clearly identified.

The authors are feminist, critical realists, with backgrounds in maternity research, global health, health systems research and anthropology, who view social reality as historically and culturally constructed and situated. Our aim for this review was to foreground the voice of the midwife, who has often been excluded from the discourse on D&A. Use of the conceptual framework allowed us to contextualise the nature and drivers of (dis)respectful care in resource-constrained environments and makes visible our interpretations and positionality.

## Results

### Search results

**Fig. 2:**
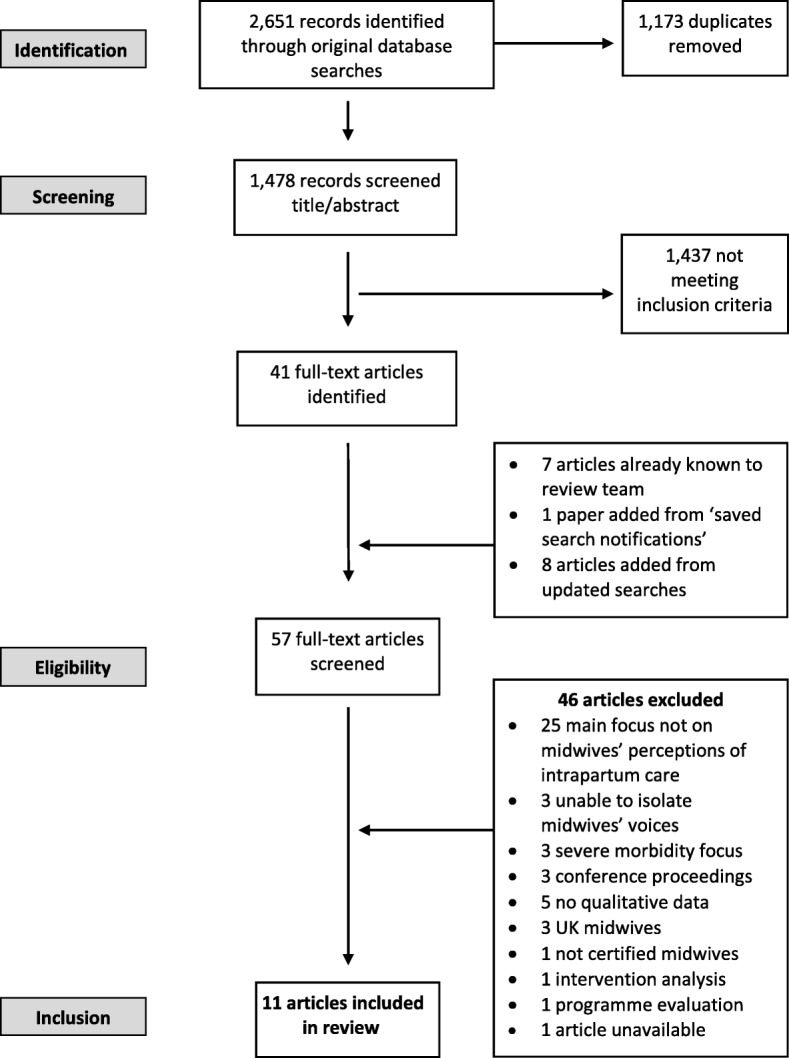
Search statistics

Electronic databases identified 2,651 papers. After title/abstract screening, 41 items were selected for full text review. A further seven papers known to the review team, one new publication from saved search notifications, and eight papers from updated searches were added (*n* = 57). The majority of excluded papers (25/46) did not have midwives’ perceptions of intrapartum care as their main focus. Others had reported medical and midwifery staff’s perceptions together, in a generic ‘health worker’ or ‘provider’ category, so did not satisfy the requirement for the midwife’s voice to be clearly identified. Other reasons for exclusion and the full search results are presented in Fig. 2.

Eleven papers were eligible for inclusion [35–45] and their study characteristics can be seen in Table 3. Two papers [39, 40] were from the same study. Quality ratings for the included papers were: one low quality; five medium quality; and five medium/high quality. The geographical spread of papers was: four from South Africa; two from each of Ghana and Mozambique; and one each from Benin, Ethiopia and The Gambia. Six papers had aims that were negatively framed: four explicitly focused on mistreatment or abuse [36, 39, 44, 45]; one looked at the psychological stress of caring [40]; and another reported midwives’ perceptions of barriers to quality perinatal care [43]. In contrast, Fujita et al. [37] reported on the implementation of a humanised care intervention. Only four explored midwives’ experiences of intrapartum care from a neutral position [35, 38, 41, 42].

**Table 3:** Characteristics of included studies

Study	First author, year	Country	Study aims	Participants^a^, setting	Study design, data collection^b^ and analysis	Quality
1.	Adolphson et al., 2016 [35]	Mozambique	• Explore midwives’ perspectives of working conditions, professional role and attitudes towards women	• 9 midwives (6 x medium, 3 x basic level) • Urban, suburban, village and remote areas in 3 southern provinces	• Qualitative methods • SSI • Content analysis	M
2.	Burrowes et al., 2017 [36]	Ethiopia	• Explore midwives’ understandings of patient rights and patient-centred care • Describe midwives’ experiences of D&A during labour and delivery and identify recommendations for improvement	• 4 midwives, 15 x BSc student midwives • Debre Markos health centres and university	• Cross-sectional qualitative • IDI • Thematic content analysis	M/H
3.	Fujita et al., 2012 [37]	Benin	• Determine how the practice of humanised care affects midwives; implementation, understanding and factors influencing change in practice.	• 6 midwives • Tertiary hospital in the capital city, Porto-Novo	• Qualitative, descriptive • IDI • Grounded theory	M
4.	Jeng, 2008 [38]	The Gambia	• Assess the practices and quality of delivery care during childbirth	• 5 midwives, 3 student midwives • Delivery ward, Royal Victoria Teaching Hospital	• Qualitative methods • IDI • Content analysis	L
5.	Kruger & Schoombee, 2010 [39] Schoombee et al., 2005 [40]	South Africa	• Explore nurses’ experiences of abuse in a maternity ward • Explore maternity nurses’ psychological and emotional experiences	• 8 ‘Coloured’^c^, middle-class, Afrikaans speaking females • Maternity ward of the local state hospital	• Social constructionist grounded theory • SSI	M/H M/H
6.	Lambert et al., 2018 [41]	South Africa	• Explore providers’ lived experiences of care during labour and birth • Inform recommendations to improve and monitor quality of care	• 30 midwives • 11 public health facilities • One urban (Guateng) and one rural (Limpopo) province	• Descriptive, phenomenological • IDI and FGD • Thematic framework analysis	M
7.	Maputle & Hiss, 2010 [42]	South Africa	• Explore and describe the experiences of midwives managing women during labour • Inform development of a woman- centered care model to be integrated into the Batho-Pele Principles	• 12 midwives • Tertiary care hospital in the Limpopo Province	• Exploratory, descriptive, contextual and inductive • IDI • Open coding (Tesch)	M
8.	Pettersson et al., 2006 [43]	Mozambique	• Explore midwives’ perception of factors obstructing or facilitating their ability to provide quality perinatal care	• 16 midwives • Labour ward, Maputo Central Hospital	• Qualitative • IDI • Grounded theory	M/H
9.	Rominski et al., 2017 [44]	Ghana	• Examine disrespectful and abusive treatment towards labouring women	• 83 final year midwifery students • 15 public midwifery training colleges in all 10 of Ghana’s regions	• Not stated • FGD • Thematic analysis	M/H
10.	Yakubu et al., 2014 [45]	Ghana	• Explore attitudes, beliefs, and self-reported behaviours of midwives to improve understanding of maltreatment during facility delivery	• 7 midwives • Small rural hospital • Central region	• Cross sectional, qualitative • SSI • Thematic analysis	M

^a^Midwifery participants only are recorded here and include midwives, nurse-midwives, advanced and student midwives

^b^Interviews – *IDI* in-depth, *SSI* semi-structured, *FGD* focus group discussion

^c^This is a problematic and contested term, but was used by the authors to describe participants

### Synthesis results

**Fig. 3:**
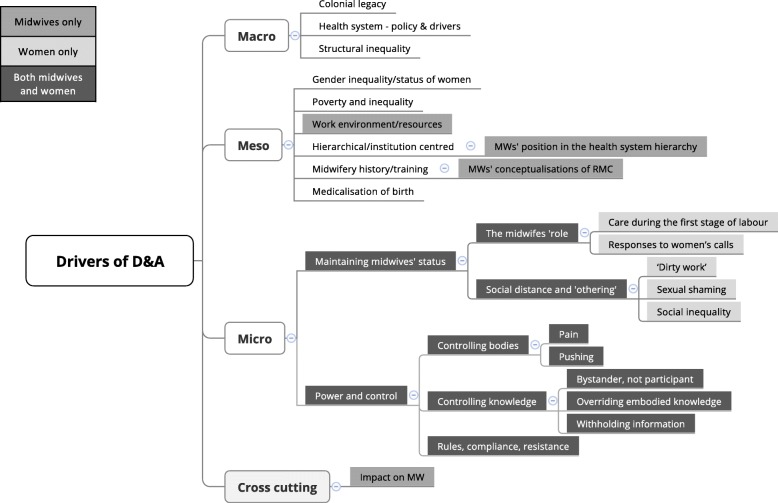
Convergence and divergence of themes arising from midwives’ and women’s experiences of (dis)respectful care

The majority of papers largely focused on the micro-level interactions between midwives and women. During the synthesis, these were mapped onto the conceptual framework’s overarching analytical themes of ‘Power and control’ and ‘Maintaining midwives’ status’ (see Fig. 1.) At the meso-level, most midwives’ focus was on immediate drivers, with ‘Work environment/resources’ a dominant theme. Other significant findings were: ‘Midwives’ position in the health system hierarchy’, a sub-theme of ‘Hierarchical/institution-centred’ health systems; and ‘Midwives’ conceptualisations of RMC’ which is nested under ‘Midwifery training/history’. The remaining meso-level themes identified in our original conceptual framework were either not mentioned (‘Poverty and inequality’) or contained insufficient data to contribute to the synthesis (‘Medicalisation of birth’; ‘Gender inequality/status of women’). Data on macro-level themes were absent from the included papers. An emerging, cross-cutting theme, which was not part of our original conceptual framework, outlined the ‘Impact on midwives’ of (dis)respectful care. Figure 3 shows the coding framework and indicates convergence and divergence between themes arising in this review of midwives’ experiences and those from our previous review of women’s experiences.

### Micro-level themes

1. **Power and control**

This theme focuses on midwives’ efforts to control women’s bodies, particularly during pushing and when women were in pain. It also shows how control of knowledge was used to gain compliance or override women’s embodied knowledge, relegating them to the position of bystander in the birth. Rules, discipline and punishment were used to exert this control.

#### Controlling bodies

The synthesis showed that controlling women’s bodies was a core component of care. This involved restrictions on what women were allowed to do, such as confining women to the bed despite knowing the benefits of ambulation, or not permitting fluid and food intake. A key trigger point, mentioned in over half the papers, was control of pushing, which Lambert et al. [41] reported as a time full of fear and raised voices. Midwives spoke of having no choice but to yell, slap or neglect women to motivate them to push, using language like ‘*need to*’ or ‘*forced to*’ when describing their actions. While some student midwives thought there was no justification for abuse, others were vocal about the necessity to use coercion to avoid bad outcomes. A quote from a student midwife typified a recurring view among participants.

*One time I was conducting a delivery and the woman was not pushing. I have said everything. I have done everything, she would not push. And I don’t know what else to do, so I just called the in-charge, she came, shouted at her some few minutes, beat her, then she started pushing. In some few minutes, the baby came out. So, if I have just left her, after explaining everything to her, I have just left her like that, the baby would have come out asphyxiated, and I could not do anything about it. So sometimes, we just have to use a little bit of force, and then they will comply.* (p.220) [44]

Another trigger point was dealing with pain and its manifestations, which some midwives described as a trigger for women to become difficult to control [40] or driving them to physically lash out [36]. However, while pain was sometimes understood as an explanation for why women did not cooperate, it did not spare them from punishment. *“Sometimes when you tell them* [women] *to do something … they would not mind you because they are in pain, so you are forced to neglect them and go and sit somewhere. Until they are willing to do what you want them to do, we will not come there.”* (p.220) [44] Conversely, the ability to alleviate pain was a source of pride in Mozambique [35], while other midwives showed empathy for women, describing them as *“often desperate with pain”* (p.153) [43] or were concerned when colleagues verbally abused adolescents during labour, *“when actually it is a time they need support, when they are in pain”* (p.400) [39]

#### Controlling knowledge

Some midwives expressed their awareness of the importance of communicating information to women [35, 37], although this could be a challenge due to time pressure [43]. Giving information was often explained as a strategy to increase the chances of women doing what they were told:

*… they must* ***just listen to what I say and do as I tell them*** *… Sometimes* ***I just leave them****, let them do their own thing, but usually I prefer for them to know …* ***so I explain to them*** *before labour what to expect and* ***how they must behave****. Then things go well. Otherwise it is a complete mess, and … and a stressful experience.* (p.394, authors’ emphasis in original paper) [40]

The idea that women did not know what to do fostered an attitude that midwives were justified in shouting or hitting them to prevent harmful behaviour [45]. Even in the study that explicitly addressed communication and where midwives viewed their role as supporting women’s decisions and participation, midwives felt that women did not have sufficient information about birth and what to expect. *“Most women who are in labour look confused and don’t listen to the instructions carefully …*” (p.9) [42]. Some expected women to just obey instructions, while others performed procedures without involving the woman.

*I am not sure how much the women in labour are informed of what is going on … e.g. why the examination is being done … how soon she might deliver … why she is being admitted … This sort of information is never given to the women … No explanation of what the women to do when they feel something … They* [nurses, midwives and doctors] *don’t tell the woman what to expect.* (p.93) [38]

However, when better communication was established with women and their families, as in the humanised birth intervention in Benin [37], midwives felt, *“It is nothing difficult or surprising”’* (p.423) and the authors reported higher motivation among midwives as a consequence.

Overriding women’s bodily knowledge was another way of exerting control over them, emphasising that authoritative knowledge over birth and its various stages was technical and institutional. A key aspect of this theme, birthing position, was mentioned in six studies [36–38, 40, 42, 43]. Position was usually treated inflexibly and dictated by midwives, even if they thought the woman should have a choice. *“Anyway, the position that the woman prefers should be adhered to … but here we* [midwives] *tell them* [woman in labour] *to lie in lithotomy position* [legs up the bed poles]*.”* (p.87) [38] Only two papers reported accommodating women’s preferred positions for birth, such as equipping midwives with the necessary skills [37] or supporting women’s choices to squat if they preferred, unless there were difficulties [42]. In the South African context, delivering on all fours was linked to socioeconomic and racial discrimination.

*Especially, and I don’t want to discriminate, but the* ***black*** *people.* [last part in whisper] *… they push on all fours. And that makes things a bit difficult because you have to be here underneath them … And it makes you a bit angry sometimes, because they … they don’t cooperate, and anything can happen if they, because they are upside down."* (p.399, emphasis in original) [40]

This uncertainty about the skills needed to safely assist a woman and the persistence of lithotomy position was supported in Fujita et al.’s [37] discussion of hesitation and difficulties in implementing humanised care. *“In the beginning, we did not have enough skills to assist freestyle birthing positions, and some perineal tears resulted. Some midwives had back pain or knee pain. But after learning through watching videos and practicing, the tears have now decreased.”* (p.423)

#### Bystander, not participant

The convergence of physical and informational control served to relegate women to the role of bystander, not participant in the birth process. This was clearly underpinned in some midwives’ accounts by a belief that the midwife knew best [42, 45] and an expectation that women should do what they were told [38, 40, 41, 43]. Respondents in Maputle and Hiss’ study [42] suggested women did not necessarily want to participate in their care, and were passive and dependent on midwives. *“Women in labour very easily put themselves into the hands of the midwives … But at times there is an attitude in our society that says a pregnant mother is ill and must leave all the responsibility to the midwives because they know best.”* (p.9) However, some felt that this was “*… because some women are from a cultural environment where the women is not used to expressing her wishes as this is not allowed.”* (p.9) In The Gambia, a midwife who reported telling women to lie in lithotomy position justified it by saying, *“I have not seen woman in labour who had ever requested for any position they want.”* (p.87) [38].

More positively, there were references for midwives to view women as participants, not bystanders, with women and midwives working together [43] or women being involved in their care [41]. *“When a woman has enough information about herself during pregnancy or delivery, she can make appropriate decisions. Our job is to help women understand themselves, and empower themselves.”* (p.424) [37].

#### Rules, compliance and resistance

Exertion of power and control over women was enforced by the use of discipline and punishment in addition to the widespread use of shouting or yelling that was normalised and often routine. Some used neglect, such as leaving women alone during labour [44] or second stage [39]. Intersecting with controlling women was the perception that certain categories were more difficult to control than others, such as black women or non-English speakers [40], while others thought some women brought disrespect upon themselves by not obeying the midwife’s instructions. *“She doesn’t want anyone to do her vagina examination so we decided to hit her so that she opens up her leg to do the V.E.”* (p.220) [44].

Discipline intersected with notions of instruction. In Ghana, some students justified physical abuse of women if they had done something wrong, such as not listening to the midwife or refusing to cooperate, but suggested the midwives should explain why, “*… so the next time the woman is coming to deliver, she will bear it in mind.”* Another student thought there was a better alternative. *“And it* [yelling] *is far, far better than the beating. So, instead of midwives beating, I think we should yell, and after yelling, you let the woman understand why you yelled at her, next time she wouldn’t repeat it again.”* (p.219) [44] The two papers based on a study in South Africa [39, 40] showed midwives using moral judgements of younger women who were pregnant to justify shouting and verbally abusing them.

In Ghana, the relationship between midwives and women was described as analogous to a mother-daughter dynamic, which manifested positively as, *“Encourage her, talk to her, be friendly with her. If you are very close to the patient … I think, she will not be naughty. Talk to her friendly, as a mother or a sister, hey this is, you can do this, you can go like this.”* However, if the woman did not comply, discipline was meted out. *“When you hit, you know it’s not right … You have to discipline her to do the right thing. So it’s a kind of discipline that we are doing.”* (p.387) [45].

2. **Maintaining midwives’ status**

This theme addresses ways in which midwives attempted to maintain their professional, technical and social status in their interactions with women. It shows the lack of attention to care during the first stage of labour, as well as the ways in which power relationships and social inequality manifested in the labour ward.

#### The midwife’s role

Some positive references were made to the midwife’s role during the first stage of labour. Two papers reported midwives showing empathy or adjusting care to the woman’s needs [35], or the importance of the initial contact [38]. However, the majority of midwives’ discussions focused on the second stage of labour. Leaving women alone during the first stage was described as standard procedure in South Africa [39] and also in Benin prior to the humanised care initiative [37], while Lambert et al. [41] noted the working definition of labour as second stage. Others attributed neglect during the first stage to staff shortages. *“They* [the women] *always want the midwife to be on their side when they are in labor. And there are only so many midwives on duty … That is why … we can’t stand by the patient until the time she delivers.”* (p.387) [45] Despite these challenges, some midwives were ambivalent about the use of labour companions [42] or suggested they could not be accommodated due to staff or infrastructural constraints [38, 41].

#### Social distance and ‘othering’ - social inequality

Comments and perceptions from four papers [36, 40, 44, 45] demonstrated ‘social inequality’ as a key driver of some of the disrespectful care meted out by midwives, where midwives discriminated against certain categories of women to decide who accessed services or how care was delivered. Interviews in a South African hospital asked midwives to articulate the psychological experience of being a maternity ward nurse, revealing a strong hierarchy of patients and how they were cared for. There were ambivalent attitudes to private patients who, on the one hand, “*… pay that little bit more than a, than a normal patient. And then they expect to have a little bit more attention or whatever …*” (p.402) [40] but, on the other, could be neglected for long periods of time if they were seen as too demanding. Adolescents were subject to scolding and moralising, while one midwife said of HIV positive patients, *“I get angry!...And and I get really, I get* ***angry*** *at* [HIV positive] *people who...*[have babies].” (p.401, emphasis in original) However, racial discrimination was the most commonly mentioned form of ‘othering’ in this context. Black patients were perceived as uncooperative or difficult, where *“...they don’t listen to you, they just do their own thing...”* (p.395) and a midwife said of her colleagues, *“No sometimes they don’t treat the blacks … the same.”* (p.399).

Student midwives in Ghana [44] were aware that poorer clients would need *“more care than ever”* but had witnessed behaviours that did not embody this. *“But the midwife did not treat her well because she …* [thought she] *was one of those women who sleep by the street.”* (p.220) In contrast, Yakubu et al. [45] concluded that maintaining social distance was not a primary motivator for midwives in their Ghana study. In Ethiopia [36], rurality, lack of education and differences in social background between women and midwives were compounded by language and communication difficulties between women and midwives.

### Meso-level themes

#### Work environment/resources

The constraints of the work environment were a dominant meso-level theme, raised by midwives in 10 of the 12 included papers. These exerted a profound impact on whether or not midwives felt they could provide respectful or quality care. Their largest concern was human resources, with nine papers specifically mentioning this. Even when there was management support for humanised care, such as in the Benin intervention, midwives still worried about the practicalities. *“When we practice humanized care, we are close to the women and families, and talk to them. I am happy to do that. But if there are too many women in the labour room, it is difficult.”* (p.426) [37] Other papers spoke of midwives’ tiredness, frustration [35] or stress [40], or how insufficient staff for the workload meant some women birthed alone or were neglected [43, 45]. Student midwives noted that being overworked and under-resourced could lead to disrespectful care. *“If you are due and they tell you to push and you are not pushing and the situation is let’s say one midwife to about five clients so if you are not ready to push it is either she hits you or something so that brings about those things.”* (p.218) [44] Others described their colleagues as lazy or unwilling to work [38, 40, 43], or suggested, *“Sometimes providers also lessen their commitment considering the low payment they get compared to their effort”*. [36]

In smaller facilities, the lack of staff meant some midwives had a broader scope of practice, which was a source of job satisfaction, but also stress [35]. However, the broad consensus was that lack of material resources, poor infrastructure and shortage of staff compromised midwives’ ability to provide the highest attainable standard of care. Midwives voiced their concerns about lack of support, with many left alone and others making requests that were not answered. *“They* [hospital administration] *are aware of this problem but when you complain to them they will tell you ‘what can we do’.”* (p.80) [38] This left some midwives feeling powerless to change their situation, either personally or collectively [43], despite their awareness of productive changes that could be made.

#### Midwives’ position in the health system hierarchy

Nested under the theme ‘Hierarchical/institution-centred’ was midwives’ perspectives on their own place in the health system hierarchy [37, 38, 40, 41, 43, 44]. This has great relevance for the overarching analytical theme of ‘Maintaining midwives’ status’. The superior status of doctors and lack of recognition for midwives’ contribution was a particular focus. Midwifery students in Ghana were aware they were not respected. *“I also think that some of the doctors especially look down on the nurses and midwives, the doctor comes to the ward they don’t even consider what you are doing, they just shout on you as if you don’t know what you are doing, you don’t know your left from your right …*” (p.219) [44] Nurse-midwives in South Africa concurred. *“But at the end when I get home, that is the worst thing that you cry alone. That, you know, everybody, they don’t see me as a qualified professional, they see me as someone who just went there. Nobody respects me because of what they think of us.”* (p.259) [41]. In Mozambique, midwives felt they had low status in the obstetric team and their opinions were ignored by physicians [43]. However, this status was temporally located. Senior staff only worked during the day, so at night midwives were trusted to make decisions. Midwives in The Gambia engaged in covert resistance when their professional judgements clashed with those of doctors who ordered them to give what midwives considered dangerous doses of Pitocin. *“Anyway you just tell them yes but you do something else.”* (p.83) [38].

The possibility for positive changes in professional relationships between cadres was described by Fujita et al. [37] in Benin. After the introduction of the humanised birth initiative, midwives expressed improved self-esteem because their professional expertise was now being recognised, and they felt more supported by obstetricians and the management team. This had constructive impacts on teamwork. *“Normal labor and delivery is our job. When a cesarean is needed or a complication happens, we work together with obstetricians. We trust obstetricians and have no problems with our relationship.”* (p.425) Only two other papers [35, 43], both set in Mozambique, mentioned the importance of teamwork, despite it being a critical element of maternal health care. Further, Lambert et al. [41] described the impact of visible, supportive leadership on midwives’ working relationships and happiness in their roles.

Difficulties in the perceived status of midwives were not just limited to their interactions with doctors. Schoombee and Kruger [40] reported numerous and complex power struggles between different grades of midwives too. These were reflected in downward behaviours such as senior midwives (sisters) scolding junior staff, but also in upward hierarchical interactions where even midwives who held positions of authority sometimes feared to exercise this and hold others to account because subordinates would blame them and initiate informal sanctions, such as withholding cooperation.

Negative hierarchical relationships in the health facility had profound implications for midwives, leaving them feeling unsupported [41] and blamed [44]. In Mozambique, midwives felt silenced by the critical nature of their interactions with the rest of the obstetric team, making them fearful of admitting any inadequacies. This negatively influenced their performance, while *“If you are scolded in front of other colleagues or even worse the laboring women, you have no authority left to perform your work.”* (p.155) [43] Elsewhere, midwives were stressed and unhappy about aggressive treatment from their colleagues, which made them fear speaking up about poor care. *“I’ve often seen that...seen a sister scold a patient. And then, I am unhappy about it, but I don’t talk about it, I keep it to myself.”* (p.402) [40].

### Midwives’ conceptualisations of RMC

This theme is nested under ‘Midwifery training/history’ in the conceptual framework and describes midwives’ understandings of RMC. Despite the strong focus on mistreatment and abuse in the majority of studies, many authors reported positive conceptualisations of RMC, particularly those who had been part of the introduction of humanised care in Benin [37]. Midwives spoke of trust and two-way communication [37, 42], treating women as individuals [44], empathy and commitment [35], always putting the patient first [40], or informing and involving them in their care [41].

Among student midwives, RMC was often conceptualised by what it was **not**. For example, one midwifery student said, *“The basic knowledge I have about respectful patient care, is irrespective of the race, the social status, the background, or whatever of the client. You...must not discriminate against them* [women] *because of who they are.”* Another stated, *“When we talk about respectful patient care I think it means caring for the patient in a respectful manner like not insulting the patient, or beating her or teasing her, you care for her emotionally and everything so that she can deliver safely.”* (p.218) [44] The perspectives of students in Ethiopia reflected a curriculum that was narrowly focused on privacy and confidentiality [36]. However, another student in Rominski et al.’s study noted the reciprocal nature of care, where both woman and midwife subjected themselves to what the other needed them to do. In a different study, there was an awareness of the contradiction between what some midwives say about respectful care and what they do. “*… few days ago we asked three midwives “what makes a midwife to be a good midwife”. All of them said that it is important to show empathy and attend to the woman’s needs and so on … They answer it but we can’t see that in them …* [she laughs].*”* (p.92) [38].

Although midwives did not use the language of professionalism when discussing (dis)respectful care, it was implicit in some descriptions of their behaviours and motivations, and was mentioned explicitly by some authors. For example, one of the overarching themes reported by Adolphson et al. [35] was ‘commitment/devotion’, with examples given purporting to reflect midwives’ hard work, independent scope of practice and pride in their work. In Pettersson et al.’s study [43], a sense of professional inadequacy and inferiority was a key thread, intersecting with the theme of ‘Maintaining midwives’ status’ in our synthesis. Two studies suggested mechanisms to improve professionalism, including recognising limitations and asking for advice [43], and introduction of humanised care [37].

### Cross-cutting theme

#### Impact on midwives

Appreciation and recognition from the community was an important factor for many midwives. “*… after the mother has pushed out she says, “Thank you for supporting us, nurse,” and every time I feel more motivated, I feel more enthusiastic.”* (p.98) [35] Others were aware of the importance of word of mouth, that respectful care and good behaviour would encourage women to come to the facility. “*… it* [RMC] *matters so much because, the attitudes of the health work* [ers] *makes the pregnant women go to the TBAs and other places.”* (p.218) [44] Providing humanised care was also reported to benefit health workers. All six midwives interviewed in Fujita et al.’s study [37] described increased satisfaction and motivation, and there were also reports of improved confidence and self-esteem. *“I am like the mama of mamas. The woman and her family trust me and ask me to attend a future delivery or tell me that they will introduce me to their friends. I am so proud of this.”* (p.424) However, concerns about the challenges of staffing and the poor working environment left some midwives feeling frustrated and inadequate when they could not provide the care they wanted to [35, 43], while Lambert et al. [41] reported midwives’ lack of role models and *‘leading by example’*. (p.259).

Some midwives described troubling negative emotions when dealing with birth, many of which were linked to their efforts to control women’s bodies. Lack of cooperation or failure to push generated angry and occasionally violent emotions [39, 40, 44]. *“Sometimes if, then the patients are difficult, they don’t want to cooperate … then you just feel … you’re not allowed to assault a patient … But sometimes you just feel like, then you think, oh, you just want to assault that patient, if the patient won’t push and so on* [strong emotion]*.”* (p.95) [39] This intersected with feelings that women, the community or line managers would hold midwives responsible for poor outcomes, regardless of the woman’s behaviour. This dynamic of blame was explicitly implicated as a driver of disrespect in two papers [44, 45]. In Ghana, the weight of responsibility meant midwives felt they needed to do ‘whatever it takes’ [45], while student midwives thought it was better to shout at or hit women than to let them fail to push or cooperate [44].

## Discussion

The primary purpose of this review was to synthesise macro-, meso- and micro-level drivers of midwives’ experiences of disrespectful care during facility-based delivery. This synthesis of midwives’ perspectives demonstrated substantial convergence with our earlier review based on women’s perceptions [14]. Controlling women was a powerful dynamic at work in the labour ward, reinforcing the message that birth was a medical event, mediated by experts. Midwives felt women did not know what to do, controlled where they could go and how they behaved, and overrode women’s embodied knowledge to dictate how women should birth. The pushing stage of labour acted as a key trigger, one of the factors which Yakubu et al. [45] called “precipitating events” for D&A. Failure to obey or transgressing the rules elicited punishments for women such as neglect, shouting and beating. Midwives, particularly students, seemed candid about D&A, supporting other literature suggesting these behaviours are normalised and widespread [46–48].

An interesting new element was midwives’ perceptions that women were intentionally ‘being naughty’, with limited empathy demonstrated for their pain or situation. This contrasts with women’s perceptions of pain as a major cause of distress and lack of control, which they expected midwives to assist with and advise upon. However, midwives’ time to support women to cope was severely constrained by staff shortages, exacerbated by resource deficits that leave pharmacological analgesia in extremely limited supply [49]. Midwives’ inability to provide pain relief and the impact of this on their sense of professionalism may well drive a dynamic of disrespectful care and bears further investigation.

This review revealed that many midwives felt driven to maintain control of women in order to avoid bad outcomes for which they would be blamed. Organisational cultures of blame intersect with ongoing staff shortages and the challenges of the “materiality of care” (including infrastructure, space and resources); these impede midwives’ ability to work professionally and have a significant impact on human interactions in the labour ward [50]. Some authors [45, 51] have suggested that one solution to address D&A would be to train midwives to be able to deal more effectively with the current constraints. However, this has the potential to push responsibility to cope back on to the midwife, when the blame for, and challenges of, the deficits of an entire health system already sit on her shoulders.

Our earlier analysis of women’s experiences had concluded that a significant driver of the behaviours midwives exhibited was an attempt to increase social distance and maintain status. This emerged less strongly when hearing directly from midwives, where only the theme of social inequality emerged. No papers mentioned the themes of sexual shaming and dirty work which had emerged from women’s accounts. This was exemplified in two papers from the same study in South Africa [39, 40] which explored both women’s and midwives’ perceptions. These had been included in our earlier meta-synthesis of women’s experiences. Women articulated significant discriminatory behaviour based on race, age and class, but this dynamic formed a smaller component of the interviews with midwives. The study from Ghana [45] suggested social distance was not an issue, describing instead a ‘mother-daughter’ relationship. This could, however, be interpreted as a way of increasing midwives’ status by infantilising women and rendering them powerless. Indeed, participants in the study likened physical abuse of women to mothers disciplining a naughty child. In postcolonial contexts, midwifery training was originally delivered by the Christian missions and was couched in terms of ‘civilising’ and offering ‘social and moral superiority’ [52, 53], characterized by the instruction and discipline that some midwives displayed in this synthesis.

Our review additionally revealed midwives’ focus on their own insecure and ambiguous position in the health system hierarchy, particularly in relation to doctors, with perceptions that midwifery was not valued. Midwives’ feelings of their professional judgement being overridden by medical staff uncomfortably mirrored their own exertion of authoritative knowledge over women’s bodily knowledge. There were also reports of hierarchical bullying between different levels of midwives. This phenomenon has been described using oppressed groups theory [54] as an explanatory mechanism in high-income midwifery contexts [55, 56], but remains relatively unexplored in the literature on midwifery in low-income contexts. However, it has significant impact for the dynamics manifesting in the labour ward. It may intersect with feelings of professionalism, which are already compromised in the challenging circumstances in which midwives operate [8, 57, 58] and which were a key issue for midwives in our review. Yet professionalism was rarely mentioned in the studies. Both professionalism and oppressed groups theory provide rich areas for future research.

With the exception of Fujita et al.’s study [37], limited awareness was demonstrated of the physiological or psychological impacts for women of (dis)respectful care. While some midwives offered examples of positive actions that constituted RMC, few spoke of why these were beneficial - for women or for themselves. Others mentioned policies that required them to accommodate choice, such as of birth position, but they feared to do so because they had only been trained for supine deliveries. Both these gaps could be addressed by more focused pre- and in-service education that provides midwives with a rationale for making change that will benefit them too, in contrast to an existing tendency to focus only on women’s rights. However, strong leadership is crucial to support and normalise respectful care in practice.

Much of the literature on D&A in sub-Saharan Africa has focussed on micro-level labour ward interactions and the results of our review reflect this. Bohren et al.’s [12] global review of mistreatment produced a typology that expanded the focus to also consider health systems factors. Freedman and Kruk [59] went further, characterizing D&A as a symptom of locally expressed power dynamics and fractured health systems (e:43). Importantly, they noted the impact of these factors on both women and health providers. Our synthesis aligns with their work as it compares midwives’ perceptions with those of women and explores the impact of (dis)respectful care on midwifery cadres at the front line of maternity care. Further, our original conceptual framework is theoretically informed, facilitating a layered and textured explanation of (dis)respectful care that extends beyond existing, descriptive frameworks of D&A (e.g. [12, 60]) to address the larger circulating discourses on how and why different actors may, or may not, abuse women. However, there was a significant lack of data relevant to the macro-level influences, such as the colonial legacy, or power and social inequalities, in the papers included in the synthesis. This is unsurprising given the immediate meso- and micro-level concerns of midwives in resource-constrained contexts. Only Rominski et al. [44] alluded to gender-based violence and the broader social and political dynamics. Kruger and Schoombee [39] discussed power and control in the context of the medical model of birth and hospital hierarchy. This left us unable to meaningfully comment on some of the broader drivers of D&A which are crucial to our understanding and efforts to improve the quality of midwifery services for both women and midwives, and are increasingly pressing as the international community strives to ensure positive intrapartum care [26]. Future research with national-level stakeholders to explore the policy, legislative, organisational and systems contexts in which midwives operate could provide a useful test of our conceptual framework’s explanatory powers at the macro-level.

## Methodological considerations

Some of the papers included in this synthesis scored well on study findings and value, but lacked detail of the methodological techniques employed [37, 39]. Others were very descriptive, lacking the conceptual richness and depth that may be necessary for interpretive synthesis [61]. In addition, the studies explored the views of midwives across a range of geographies, cadres, and levels of care, but most provided insufficient detail to allow us to explore the influence of rurality, level of qualification or level of institution on the findings. A further limitation was that over half the papers did not demonstrate any attention to reflexivity. While for some authors this may have been due to journal space constraints, it is nonetheless an important issue when discussing sensitive issues such as disrespectful care. For example, in two studies where midwives were more positive about their role and behaviour [35, 37] the data were collected by doctors, which raises questions about social desirability bias affecting participants’ responses.

The majority of included studies were from countries formerly colonised by the British, Portuguese and French. Each colonial power left its own legacy, so results cannot be generalised. However, while our focus was on sub-Saharan Africa, D&A can be seen as a manifestation of structural violence [62, 63], reflecting broader gender and power inequalities that are not limited to postcolonial settings. Our conceptual framework can be modified for use in other contexts, as it provides a sufficiently flexible tool to interrogate the macro-level causes of D&A, as well as the micro- and meso-level symptoms which affect women - both those giving birth as well as those who attend them.

## Conclusion

Significant convergence was seen between the themes arising in this synthesis of midwives’ perceptions and those derived from women’s experiences in our earlier review. This was most apparent at the micro-level, where both groups described midwives’ control of women’s bodies. Pain and pushing acted as trigger points for D&A and intersected with midwives’ fear of blame. However, midwives showed less awareness of the social distance and othering that caused women such distress, instead focussing on their own low status within the health system hierarchy and the challenges of the severely constrained contexts in which they work. Many of the challenges in the labour ward that drive D&A or block RMC are contingent upon the historical, cultural and health systems factors prevailing in the postcolonial context. Our conceptual framework provides a theoretically informed basis for interrogating these factors, avoiding a micro-level focus and generating a more nuanced understanding of the broader context in which midwives’ behaviour is embedded. Lack of understanding for these professionals and the constraints under which they operate sells midwives and their efforts short, effectively constituting D&A of midwives. Serious consideration of the legacies that have shaped the health system, such as models of care and training, and the prevailing cultural norms within which these are nested, is vital. This will necessitate much wider, interdisciplinary perspectives to find meaningful and respectful ways of consulting with midwives, women and communities to address the challenges they face together.

## Data Availability

Interested parties can obtain supporting data by contacting the corresponding author.

## Abbreviations

D&A: Disrespect and abuse
FGD: Focus group discussion
IDI: In-depth interview
RMC: Respectful maternity care
SSI: Semi-structured interview
WHO: World Health Organization

## Footnotes

1 Authoritative knowledge is the knowledge that ‘counts’ within a particular social environment (for example, a health facility) and which forms the basis for decision-making and action within that environment [32].

2 ‘Dirty work’ involves working with bodily fluids and is usually seen as culturally low in status. Sociological literature on ‘dirty work’ has long described attempts by various healthcare professions to delegate such work to those of lesser status [33, 34].
